# Star-PAP, a poly(A) polymerase, functions as a tumor suppressor in an orthotopic human breast cancer model

**DOI:** 10.1038/cddis.2016.199

**Published:** 2017-02-02

**Authors:** C Yu, Y Gong, H Zhou, M Wang, L Kong, J Liu, T An, H Zhu, Y Li

**Affiliations:** 1 State Key Laboratory of Phytochemistry and Plant Resources in West China, Kunming Institute of Botany, Chinese Academy of Sciences, Kunming, China; 2 University of Chinese Academy of Sciences, Beijing, China

## Abstract

Star-PAP is a noncanonical poly(A) polymerase and required for the expression of a select set of mRNAs. However, the pathological role of Star-PAP in cancer largely remains unknown. In this study, we observed decreased expression of Star-PAP in breast cancer cell lines and tissues. Ectopic Star-PAP expression inhibited proliferation as well as colony-forming ability of breast cancer cells. In breast cancer patients, high levels of Star-PAP correlated with an improved prognosis. Moreover, by regulating the expression of BIK (BCL2-interacting killer), Star-PAP induced apoptosis of breast cancer cells through the mitochondrial pathway. The growth of breast cancer xenografts in NOD/SCID mice was also inhibited by the doxycycline-induced Star-PAP overexpression. Furthermore, Star-PAP sensitized breast cancer cells to chemotherapy drugs both *in vitro* and *in vivo*. In mammary epithelial cells, Star-PAP knockdown partially transformed these cells and induced them to undergo epithelial–mesenchymal transition (EMT). These findings suggested that Star-PAP possesses tumor-suppressing activity and can be a valuable target for developing new cancer therapeutic strategies.

Breast cancer is the most frequently diagnosed cancer and the leading cause of cancer death in women worldwide, with an estimated 1 676 600 new cases and 521 900 deaths according to GLOBOCAN 2012.^1^ Cancer cells, including breast cancer cells, usually manifest aberrant gene expression that leads to malignancy, such as sustaining proliferation, metastasis and evasion of apoptosis.^2^ Identification of the deregulated gene expression may advance the understanding of cancer pathogenesis and provide insights into potential new therapeutic strategies.

Recent advances reveal mRNA 3′end processing as a highly regulated process and an important mechanism for posttranscriptional gene expression.^3^ This process controls stability, translocation and translation of mRNAs and plays a critical role in human physiology and pathology, including cancer.^4^ Star-PAP, a noncanonical poly(A) polymerase encoding by gene *TUT1*, is a component of the 3′end processing complex required for a subset of pre-mRNAs.^5, 6, 7^ Star-PAP regulates the expression of target genes involved in various cellular processes, such as oxidative response and apoptosis, through processing their mRNAs.^8, 9, 10^ However, the physiological and pathological roles of Star-PAP largely remain unknown. Recently, it was reported that Star-PAP inhibits the lipogenesis and thus the cell proliferation in osteosarcoma cells *in vitro*.^11^ However, the functional role of Star-PAP in cancer development and treatment is unclear.

Given the clues above, the function of Star-PAP in human breast cancer was investigated in this study. We observed that breast cancer manifests a downregulated expression of Star-PAP. Ectopic expression of Star-PAP inhibited the proliferation of breast cancer cells, and Star-PAP induced the mitochondrial apoptosis through regulating the expression of BIK (BCL2-interacting killer). Moreover, Star-PAP relieved breast cancer progression *in vivo* and sensitized breast cancer cells to chemotherapeutics. This study indicated that Star-PAP has a potential tumor-suppressing function in breast cancer and can be a valuable molecular target for breast cancer therapy and prevention.

## Results

### Star-PAP is downregulated in breast cancer

**Figure 1:**
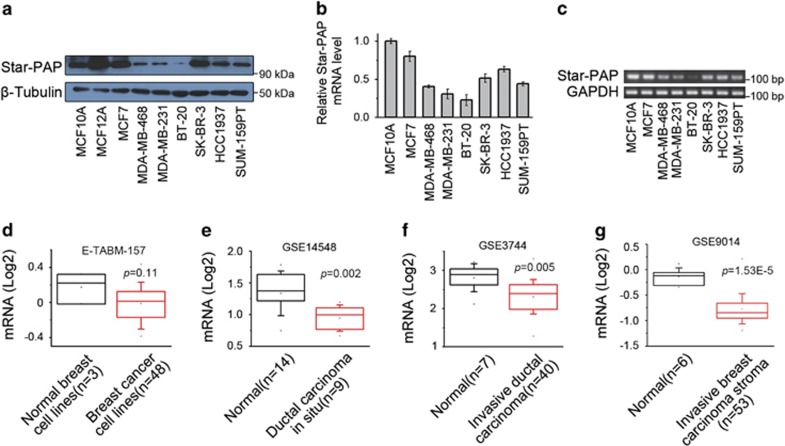
Star-PAP is downregulated in breast cancer. (**a**) Protein levels of Star-PAP in a panel of breast cancer cell lines were detected by western blot. *β*-Tubulin was used as the loading control. (**b**) mRNA levels of Star-PAP were quantified by qPCR and normalized to MCF10A cells. Data from triplicate experiments were presented as mean±S.D. (**c**) Star-PAP mRNA was examined by RT-PCR and the products were analyzed in agarose gel. GAPDH was used as the loading control. (**d**) Expression levels of Star-PAP in 51 breast cell lines were analyzed using the Neve cell line data set. Data set accession number and *P-*value are shown. (**e–g**) Box plots show Star-PAP expression levels in human breast cancer tissues. Data sets for ductal carcinoma *in situ* (**e**), invasive ductal carcinoma (**f**) and invasive breast carcinoma stroma (**g**) were analyzed. Data set accession number and *P-*value are shown

To investigate whether Star-PAP involves in human breast cancer pathogenesis, we first examined the expression of Star-PAP in a panel of breast cancer cell lines. When compared with the untransformed mammary epithelial cells (MCF10A and MCF12A), breast cancer cells showed downregulated protein levels of Star-PAP, determined by western blot (Figure 1a). Breast cancer cells also had reduced mRNA levels of Star-PAP as shown by qPCR (Figure 1b) and further confirmed by RT-PCR (Figure 1c). We further analyzed the expression pattern of Star-PAP in Oncomine, a publicly accessible cancer informatics database, with the Neve cell line data set that includes the transcriptional profiles of 51 breast cell lines.^12, 13^ Among this collection of cell lines (*n*=51), the breast cancer cell lines (*n*=48) also exhibited lower Star-PAP expression levels than the immortalized breast epithelial cell lines (*n*=3; HBL-100, MCF10A, MCF12A) (Figure 1d). Collectively, the above results indicated that the downregulation of Star-PAP is a manifestation of breast cancer cells.

Next, the expression of Star-PAP in human breast cancer tissues was examined by analyzing the public data sets.^14, 15, 16^ Although highly expressed in normal mammary tissues, Star-PAP was downregulated in ductal carcinoma *in situ* (Figure 1e), invasive ductal carcinoma (Figure 1f) and even the stroma of invasive ductal carcinoma (Figure 1g). These data proved that the expression of Star-PAP is also downregulated in clinical breast cancer progression, thus suggesting that Star-PAP may play a potential tumor-suppressing role in breast cancer.

### Star-PAP inhibits proliferation of breast cancer cells and correlates with breast cancer prognosis

**Figure 2:**
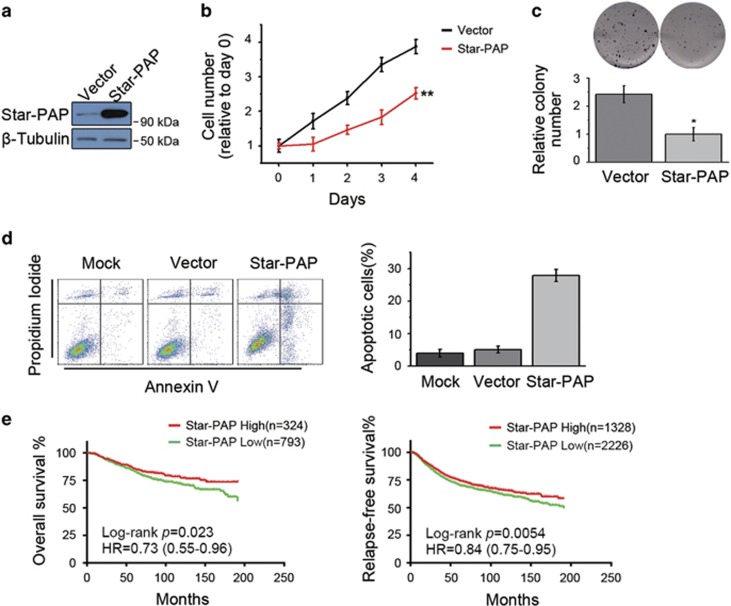
Star-PAP inhibits proliferation of breast cancer cells and correlates with prognosis of patients. (**a**) Ectopic expression of Star-PAP in MCF7 cells was examined by western blot. *β*-Tubulin was used as the loading control. (**b**) Star-PAP inhibited proliferation of MCF7 cells. Cells were plated after transfection. Proliferation of cells was analyzed by MTS assay. Data were normalized to day 0; *n*=6, **P<0.01. (**c**) MCF7 cells transfected with pCDNA3.1-Star-PAP were plated at 5000 cells/well, and colonies were stained with crystal violet. Representative images are shown (upper panel), and data from two replicate experiments were normalized and presented as mean±S.D. (lower panel). *P<0.05. (**d**) Star-PAP induced cell apoptosis of MCF7 cells (left panel), and data from triplicate experiments are presented as mean±S.D. (right panel). (**e**) Kaplan–Meier analysis of overall survival (left panel) and relapse-free survival (right panel) of breast cancer patients stratified by the expression levels of Star-PAP. Number of patients, log-rank *P-*value and hazard ratio (HR) are shown

To elucidate the functional role of Star-PAP in breast cancer progression, we first overexpressed Star-PAP in MCF7 and SUM-159PT cells. In both breast cancer cell lines, the cell proliferation (Figure 2b and Supplementary Figure S1c) as well as the colony-forming ability (Figure 2c and Supplementary Figure S1b) was inhibited by the increased expression levels of Star-PAP (Figure 2a and Supplementary Figure S1a). Moreover, ectopic expression of Star-PAP also induced apoptosis of cancer cells (Figure 2d). Together, these findings indicated that Star-PAP functions as a possible suppressor for breast cancer cells.

We then exploited KM-plotter, an online cancer survival analysis database,^17^ to investigate the correlation between Star-PAP levels and clinical prognosis. When evaluated across the entire spectrum of breast cancer patients, high levels of Star-PAP were associated with relatively increased overall survival and relapse-free survival (Figure 2e). When looking into each of the four major breast cancer subtypes (basal-like, her2-enriched, luminal A and luminal B), elevated levels of Star-PAP also indicated an improved relapse-free survival, especially in basal-like and her2-enriched subtype (Supplementary Figures S2a–d). Thus, in breast cancer patients, high levels of Star-PAP correlated with an improved prognosis.

### Star-PAP induces apoptosis through regulating BIK expression

**Figure 3:**
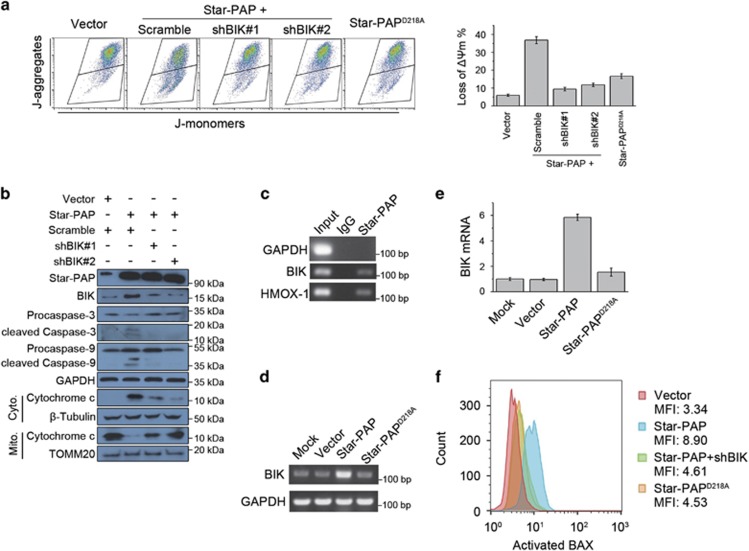
Star-PAP induces mitochondrial apoptosis through regulating expression of BIK. (**a**) Loss of *ΔΨ*m in SUM-159PT cells was measured by flow cytometry using JC-1 dye after being transfected with pCDNA3.1-Star-PAP and BIK shRNA/scramble, or Star-PAP^D218A^ (left panel). Data from triplicate experiments were presented as mean±S.D. (right panel). (**b**) Cell lysates from SUM-159PT cells transfected with indicated plasmids were analyzed by western blot. For analyzing cytochrome *c*, mitochondrial and cytosolic fractions were separated. GAPDH was used as the loading control for whole cell lysates. *β*-Tubulin and TOMM20 were used as the cytosolic and mitochondrial loading control, respectively. (**c**) BIK mRNA was detected by RT-PCR after RIP of Star-PAP in SUM-159PT cells. GAPDH and HMOX-1 were used as the negative and positive control, respectively. (**d**) SUM-159PT cells were transfected with pCDNA3.1-Star-PAP or Star-PAP^D218A^, and BIK mRNA was examined by RT-PCR 24 h later. GADPH was used as the loading control. (**e**) After ectopic expression of Star-PAP or Star-PAP^D218A^ in SUM-159PT cells, change of BIK mRNA level was quantified by qPCR and normalized to mock; *n*=3, error bar denotes S.D. (**f**) Activation of BAX was measured by flow cytometry using the conformation-specific antibody after being transfected with the indicated plasmids. Mean of MFI (mean fluorescence intensity) from two independent experiments is shown

Considering that Star-PAP induced apoptosis in breast cancer cells (Figure 2d), we then investigated the mechanism underlying the apoptosis-inducing activity of Star-PAP. We observed that Star-PAP overexpression caused approximately fivefold loss of mitochondrial transmembrane potential (*ΔΨ*m) than control (Figure 3a). Moreover, ectopic Star-PAP expression triggered the release of cytochrome *c*, a mitochondrial intermembrane space protein, and increased the activation of downstream Caspase 9 and Caspase 3 (Figure 3b). As the mitochondrial pathway of apoptosis manifests the loss of *ΔΨ*m and the release of cytochrome *c*,^18^ the above results suggested that Star-PAP induces apoptosis of breast cancer cells through the mitochondrial pathway.

The mitochondrial pathway of apoptosis is tightly regulated by the BCL-2 protein family through the interaction between its subgroup members. BIK is an inducer of mitochondrial apoptosis by activating BAX.^19, 20, 21, 22, 23^ It has been reported that Star-PAP associates with BIK mRNA and thus facilitates BIK expression by processing its mRNA 3′end in HEK293 cells.^9^ For this reason, we speculated that the Star-PAP-induced mitochondrial apoptosis of breast cancer cells is mediated by BIK. In human breast cancer cells, the association between Star-PAP and BIK mRNA was firstly verified by RNA immunoprecipitation (RIP) followed by RT-PCR (Figure 3c). Moreover, Star-PAP increased both mRNA (Figures 3d and e) and protein levels (Figure 3b) of BIK. Therefore, these results indicated that Star-PAP regulates the expression of BIK in the context of breast cancer cells.

Targeting BIK with two distinct shRNAs (shBIK#1, shBIK#2) effectively relieved the loss of *ΔΨ*m triggered by Star-PAP overexpression (Figure 3a). The release of cytochrome *c* and the activation of Caspase 9 and Caspase 3 were also effectively inhibited by BIK knockdown (Figure 3b). As a consequence of the increased BIK level after Star-PAP overexpression, the activation of BAX was also elevated (Figure 3f), whereas BIK knockdown mostly counteracted this alteration (Figure 3f). It is reported that Star-PAP^D218A^, a polymerase-dead mutant of Star-PAP, loses the poly(A) polymerase activity as well as the ability to control the expression of BIK.^9^ We found that Star-PAP^D218A^ failed to induce the loss of *ΔΨ*m (Figure 3a) and the activation of BAX (Figure 3f), thus suggesting that the polymerase activity of Star-PAP is necessary for its apoptosis-inducing ability that is mediated by BIK. Collectively, these results suggested that Star-PAP, by regulating the expression of BIK, induces apoptosis of breast cancer cells through the mitochondrial pathway.

### Star-PAP inhibits the progression of breast cancer *in vivo*

**Figure 4:**
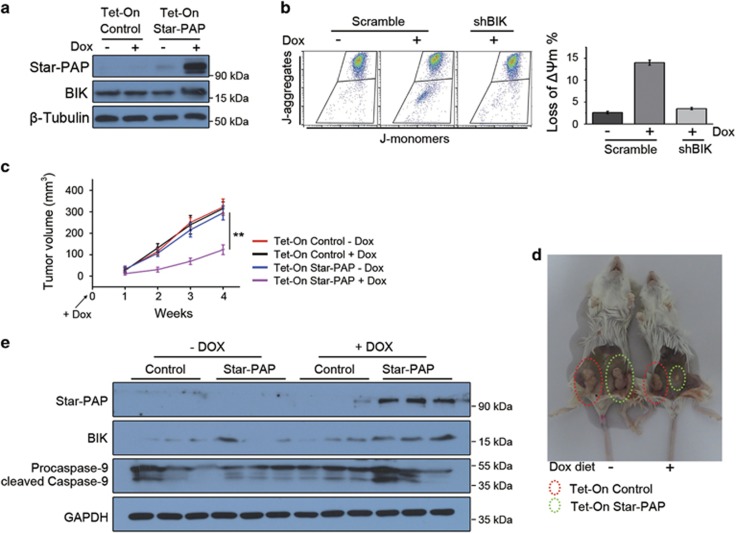
Star-PAP inhibits progression of breast cancer *in vivo*. (**a**) MDA-MB-468 Tet-On Star-PAP cells were induced with 200 ng/ml doxycycline for 24 h. Cell lysates were detected for Star-PAP and BIK by western blot. *β*-Tubulin was used as the loading control. (**b**) Loss of *ΔΨ*m in Tet-On Star-PAP cells was analyzed by JC-1 assay after treatment with doxycycline (left panel). Data from triplicate experiments were presented as mean±S.D. (right panel). (**c**) The same amount of Tet-On control and Star-PAP cells was separately incubated into the mammary fat pad of NOD/SCID mice, and one group of mice was supplied with diet containing 0.02% doxycycline immediately. Tumor volume was measured weekly and presented as mean±S.D.; *n*=8, **P<0.01. (**d**) Mice bearing xenografts were necropsied at the end of experiment. Representative mice are shown. (**e)** Lysates from several xenografts were examined by western blot. GAPDH was used as the loading control. Dox, doxycycline

To clarify the influence of Star-PAP on breast cancer *in vivo*, by stably transfecting MDA-MB-468 cells with a Tet-On plasmid encoding Star-PAP, we established a cell line with doxycycline-inducible Star-PAP expression. In the presence of doxycycline, highly expressed Star-PAP was induced and, consequently, the elevated expression of BIK was also detected (Figure 4a and Supplementary Figure S3a). As observed above, in MDA-MB-468 cells, the doxycycline-induced Star-PAP overexpression also inhibited cell proliferation (Supplementary Figure S3b) and provoked cell apoptosis (Supplementary Figures S3c and d). Moreover, the apoptosis of cells induced by the doxycycline-controlled Star-PAP overexpression also depended on BIK (Figure 4b).

We then incubated the Tet-On Star-PAP cells into the fourth inguinal mammary fat pad of NOD/SCID mice and the same amount of Tet-On control cells into the contralateral mammary fat pad. The mice were immediately supplied with the diet containing doxycycline. Tumor volume was measured weekly, and mice were killed for necropsy at the end of experiment. As revealed by tumor volume and necropsy, the growth of breast cancer xenografts was significantly inhibited by the induced overexpression of Star-PAP (Figures 4c and d). As shown by western blot using the lysates from tumor xenografts, the observed inhibition of tumor was probably caused by the BIK-mediated cell apoptosis (Figure 4e). In conclusion, Star-PAP inhibited the progression of breast cancer *in vivo*.

### Star-PAP sensitizes breast cancer cells to chemotherapy drugs

**Figure 5:**
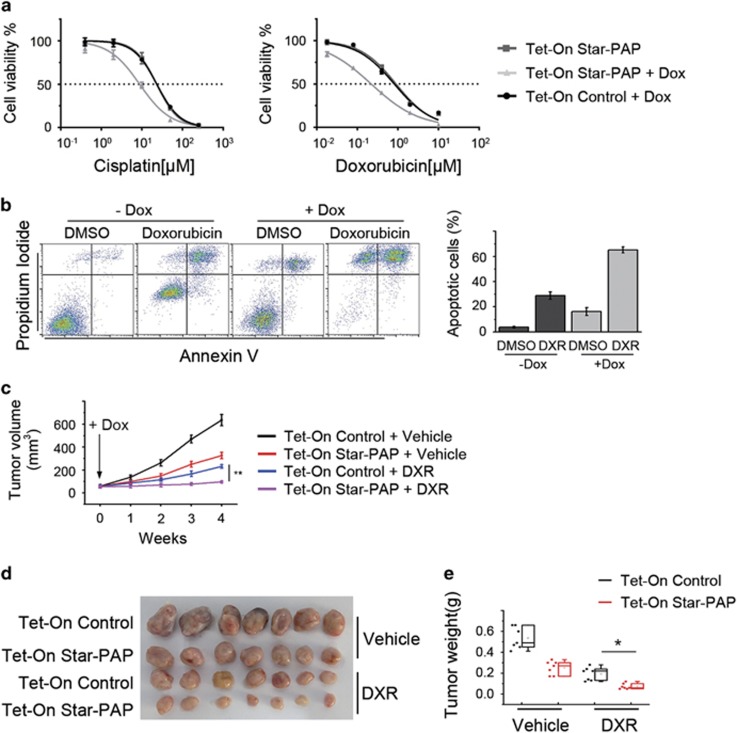
Star-PAP sensitizes breast cancer cells to chemotherapy drugs. (**a**) Dose–response curves of Tet-On Star-PAP cells treated with cisplatin (left panel) and doxorubicin (right panel). Cell viability was measured by MTS assay; *n*=6, error bar denotes S.D. (**b**) Apoptosis of Tet-On Star-PAP cells treated with 1 *μ*M doxorubicin was analyzed in the absence or presence of doxycycline (left panel). Data from triplicate experiments are presented as mean±S.D. (right panel). (**c**) The same amount of Tet-On control and Star-PAP cells were incubated into the left and right mammary fat pad of NOD/SCID mice, respectively. Mice bearing xenografts were separated into two groups and treated with vehicle or doxorubicin, and diet containing doxycycline was supplied along with treatment. Tumor volume was measured weekly and presented as mean±S.D.; *n*=8, **P<0.01. (**d**) Image of representative xenografts is shown. (**e**) Box plot shows tumor weight. Data were presented as mean±S.D.; *n*=8. Dox, doxycycline; DXR, doxorubicin. *P<0.05

The initiation of mitochondrial apoptosis is a key step that determines the effect of various chemotherapeutic agents and the drug sensitivity of cancer cells.^24, 25^ As shown above, Star-PAP induces apoptosis in the mitochondrial pathway, and therefore we investigated whether Star-PAP overexpression affects the drug sensitivity of breast cancer cells. In MDA-MB-468 cells, doxycycline-induced upregulation of Star-PAP conferred drug sensitivity to cisplatin (~3-fold decrease in IC_50_) as well as doxorubicin (~4-fold decrease in IC_50_) (Figure 5a) and consequently increased the doxorubicin-induced apoptosis (~35% increase) (Figure 5b). Similarly, ectopic expression of Star-PAP sensitized SUM-159PT cells to cisplatin (~2.5-fold decrease in IC_50_) and doxorubicin (~2-fold decrease in IC_50_) (Supplementary Figures S4a and b) and thus augmented the apoptosis caused by doxorubicin (~30% increase) (Supplementary Figures S4c and d). These results implied that increased Star-PAP levels may improve the effect of chemotherapeutics on tumor *in vivo*.

This notion was then verified by using Tet-On Star-PAP tumor xenografts in NOD/SCID mice. When the tumors were palpable, doxorubicin was administered, and diet containing doxycycline was supplied at the beginning of treatment. As evaluated by the tumor volume (Figure 5c) and weight (Figures 5d and e), the therapeutic efficacy of doxorubicin was markedly improved by overexpression of Star-PAP. Taken together, these findings indicated that Star-PAP sensitizes breast cancer cells to chemotherapy drugs both *in vitro* and *in vivo*.

### Star-PAP knockdown partially transforms mammary epithelial cells

**Figure 6:**
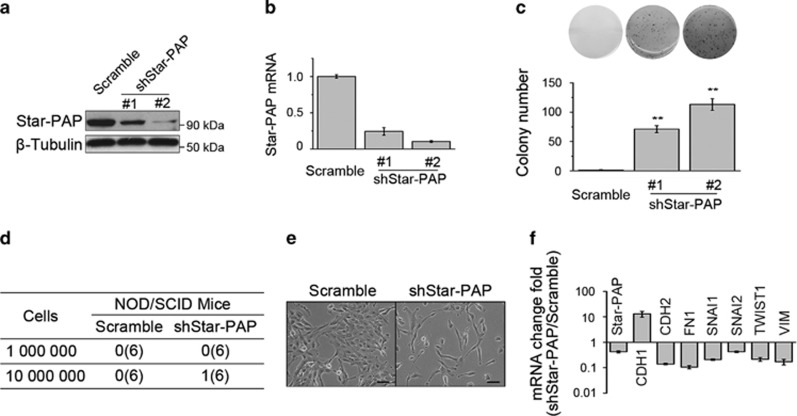
Star-PAP knockdown partially transforms mammary epithelial cells. (**a** and **b**) Stable knockdown of Star-PAP in MCF10A cells was examined by western blot and qPCR. Cell lysates and mRNA from two different clones were used. *β*-Tubulin was used as the loading control. mRNA levels were normalized to scramble and presented as mean±S.D. (*n*=3). (**c**) The anchorage-independent growth ability of two Star-PAP knockdown MCF10A cell clones was determined by soft agar colony-formation assay. Representative images are shown (upper panel), and colony number was plotted (lower panel); *n*=4, **P<0.01. (**d**) Control and Star-PAP knockdown cells were incubated in the right and left mammary fat pad of NOD/SCID mice according to the indicated cell number, respectively. Tumor was inspected 12 weeks later. Six mice were used for each group. (**e**) Representative microscopy images are shown for control and Star-PAP knockdown cells. Scale bar represents 100 *μ*m. (**f**) Change of EMT markers was detected by qPCR. Fold of change relative to scramble is shown; *n*=3, error bar denotes S.D.

Inactivation or loss of tumor suppressor predisposes normal cells to the acquisition of oncogenic transformation. We had shown that Star-PAP has potential tumor-suppressing activity in breast cancer, and then we wondered whether Star-PAP knockdown transforms normal breast cells. To determine the role of Star-PAP in mammary epithelial cells, we first knocked down Star-PAP in untransformed breast cell MCF10A using two independent siRNAs (siStar-PAP#1, siStar-PAP#2). When introduced separately into MCF10A cells, both of these siRNAs evidently decreased the levels of Star-PAP (Supplementary Figures S5a and b) and significantly promoted proliferation of cells (Supplementary Figure S5c). We generated the stable Star-PAP knockdown cells by lentivirus-mediated transfection of shRNA. As determined by soft agar colony-formation assay (Figure 6c) using two different clones (shStar-PAP#1, shStar-PAP#2; Figures 6a and b), these cells acquired the ability of anchorage-independent growth after Star-PAP knockdown. However, when incubated in the mammary fat pad of NOD/SCID mice, Star-PAP knockdown MCF10A cells failed to form tumors (Figure 6d). In addition, as shown by the change of cell morphology (Figure 6e) and the upregulation of mesenchymal markers (Figure 6f), Star-PAP knockdown induced MCF10A cells to undergo the process of epithelial–mesenchymal transition (EMT). Collectively, these data suggested that Star-PAP knockdown partially transforms the mammary epithelial cells.

## Discussion

Star-PAP is a noncanonical poly(A) polymerase that controls the expression of a subset of genes involved in various cellular processes.^6^ However, the physiological and pathological roles of Star-PAP largely remain unknown. Here, we have shown that breast cancer manifests reduced Star-PAP expression and high Star-PAP level implies a better prognosis for breast cancer patients. By regulating BIK expression, Star-PAP induced apoptosis of cancer cells through the mitochondrial pathway, inhibited progression of breast cancer and sensitized breast tumors to chemotherapeutic drugs. This study proved that Star-PAP has a potential tumor-suppressing role in breast cancer pathogenesis.

Star-PAP is encoded by gene *TUT1* that is ubiquitously expressed in normal human tissues and has putative family members from *Schizosaccharomyces pombe* to *homo sapiens.*^6, 8^ In this study, we examined the expression of Star-PAP, both mRNA and protein levels, in breast cancer cells as well as clinical breast tumors, and we observed a consistent differential Star-PAP expression existing between the normal and cancerous breast condition (Figure 1). Recently, the downregulation of Star-PAP has also been observed in osteosarcoma, the most common type of bone cancer, by PCR microarray using cDNA from clinical cancer tissues.^11^ These evidences suggest that a consistent dysregulation of Star-PAP may exist in various cancer types. However, in order to comprehensively define the functional role of Star-PAP in human cancer, more investigations should be conducted in other tumor types.

In breast cancer cells, Star-PAP induced apoptosis through the mitochondrial pathway that is tightly regulated by the BCL-2 protein family. It has been established that Star-PAP controls the expression of BIK, a member of the BCL-2 family proapoptotic proteins,^18, 26^ through regulating 3′end processing of its mRNA in HEK293 cells.^9^ Therefore, we inspected the relation between Star-PAP and BIK in breast cancer cells (Figure 3c). We revealed that by regulating the expression of BIK, Star-PAP acquires the ability to inhibit growth of breast cancer cells through the mitochondrial apoptosis pathway (Figures 3a–e). We also observed that the apoptosis-inducing ability of Star-PAP is abolished by BIK knockdown (Figures 3a, b and f). Therefore, in breast cancer cells, the apoptosis induced by Star-PAP overexpression is dependent on BIK. Given the fact that BIK is just one of the many target genes of Star-PAP, this finding gives a hint that Star-PAP may fulfill various physiological and pathological roles through its different target genes. In this study, we proved that Star-PAP, by regulating expression of BIK, induces apoptosis of breast cancer cells through the mitochondrial pathway. Recently, several reports have suggested that the function of BIK goes beyond the regulation of apoptosis. Through changing its subcellular localization, BIK is involved in response to oxidative stress caused by chemotherapy in several breast cancer cell lines.^27^ Moreover, BIK controls the expression of miRNAs as well as the autophagic flux in MDA-MB-231 cells.^28^ Thus, it would be interesting to investigate whether Star-PAP is involved in these new functions of BIK.

The mitochondrial apoptosis pathway is commonly deregulated in cancer cells and plays a key role in cancer development.^29, 30^ Mitochondrial apoptosis is also a key factor that determines the effect of various chemotherapeutic agents and the drug sensitivity of cancer cells.^24, 31^ Because of the fact that BIK is an inducer of mitochondrial apoptosis, activating BIK has been used as an anticancer strategy. However, it is a challenge to drug BIK by chemical compounds at the present time. Several alternative ways to increase BIK expression have been established, such as vector-mediated BIK expression,^32^ adenovirus-mediated BIK expression and BIK-based gene therapy.^33, 34, 35, 36^ By promoting the polyadenylation of BIK mRNA, Star-PAP increases BIK expression and induces mitochondrial apoptosis of cancer cells (Figure 3b), indicating that Star-PAP overexpression may sensitize cancer cells to chemotherapy drugs. In this study, utilizing breast cancer xenografts in NOD/SCID mice, we showed that ectopic overexpression of Star-PAP significantly inhibits cancer progression and sensitizes tumor to doxorubicin *in vivo* (Figures 4 and 5). Although Star-PAP indicated an improved prognosis in breast cancer patients (Figure 2 and Supplementary Figure S2), it is not as good as in the xenografts. We noticed that when Star-PAP was ectopically overexpressed, the levels of Star-PAP increased ~20-fold in xenografts (Figure 4e). However, breast cancer patients have a low expression level of Star-PAP, and even the patients defined as Star-PAP-high group just have a slightly higher Star-PAP level than the Star-PAP-low group. This may be the reason for the difference mentioned above.

Moreover, Star-PAP^D218A^, a mutant without poly(A) polymerase activity, lost the capacity of increasing BIK expression and failed to induce cell apoptosis, demonstrating that the polymerase activity of Star-PAP is necessary for the expression of BIK and the apoptosis-inducing role of Star-PAP (Figures 3a and d–f). Considering that the polymerase activity of Star-PAP can be activated by chemical compounds, the enzyme activators of Star-PAP may indirectly augment BIK expression and thus facilitate BIK-based anticancer strategy for clinical treatments. Therefore, Star-PAP is a valuable target for drug discovery.

Although both *in vitro* and *in vivo* evidences indicated that Star-PAP serves as a potential tumor-suppressing protein, knock down of Star-PAP only partially transformed mammary epithelial cells (Figure 6). The Star-PAP knockdown MCF10A cells acquired the anchorage-independent growth ability, a crucial step in the acquisition of malignancy,^37^ but failed to produce tumors in NOD/SCID mice (Figures 6c and d). These data suggested that partial loss of Star-PAP is not enough for the initiation of breast cancer, and also indicated that some other genetic alteration may be required to collectively complete the process of oncogenesis. Moreover, Star-PAP knockdown induced EMT, a central driver of tumor malignancy,^38, 39, 40^ in mammary epithelial cells (Figures 6e and f). It will be interesting to investigate how the lack of Star-PAP expression influences EMT and tumorigenesis.

In conclusion, we discovered the tumor-suppressing activity of Star-PAP in human breast cancer. Star-PAP is downregulated in breast cancer and correlated with prognosis of breast cancer patients. Star-PAP induces apoptosis of breast cancer cells through the mitochondrial pathway, inhibits breast cancer progression and sensitizes breast cancer to chemotherapy drugs. These findings advanced the understanding of breast cancer pathogenesis and suggested an alternative way to augment BIK expression for improving the clinical outcome of cancer chemotherapy treatments.

## Materials and Methods

### Cells and transfection

All cell lines except SUM-159PT were obtained from ATCC (Manassas, VA, USA) and authenticated by short tandem repeat profiling. All cell lines were mycoplasma free and passaged no more than 6 months after resuscitation. SUM-159PT was obtained from Asterand (Hertfordshire, UK) and cultured in Ham's F12 medium supplemented with 5% FBS, 1 *μ*g/ml hydrocortisone and 5 *μ*g/ml insulin. All other cell lines were cultured according to the ATCC instructions. Lipofectamine 3000 (ThermoFisher, Waltham, MA, USA) was used for both siRNA and plasmid transfection according to the manufacturer's instructions.

### Plasmids, siRNAs and lentivirus

Star-PAP cDNA (Genecopoeia, Rockville, MD, USA) was cloned into pCDNA3.1(+) vector for transient overexpression. For Tet-On inducible expression, Star-PAP was cloned into pLVX-TRE3G response vector (Clontech, Mountain View, CA, USA), and then the lentivirus production and infection was conducted according to the manufacturer's instructions. For Star-PAP knockdown, two previously reported siRNAs were used,^6^ and a shRNA sharing the same target sequence with siRNA#1 was used to generate stable cell line. The shRNAs for BIK were derived from TRC library database (GPP web portal, Broad Institute, Cambridge, MA, USA) and cloned into pLKO.1-TRC vector (Addgene, Cambridge, MA, USA), and lentivirus production and infection was conducted according to the TRC protocols.

### Cell proliferation assay

For cell proliferation assay, SUM-159PT and MCF7 cells were trypsinized and replated into 96-well plates at the concentration of 2000 cells/well in triplicate after transfection. Absorbance was firstly measured using CellTiter 96 (Promega, Madison, WI, USA) 3 h later, and then another measuring was conducted every 24 h.

### Colony-formation assay

Colony-formation assay was performed as previously described.^41^ Briefly, 5000 cells were trypsinized and seeded in six-well plates after transfection, and medium was changed twice every week. Colonies were stained with 0.2% crystal violet 2 weeks later and imaged for analyzing.

### Western blot and antibodies

Cells were lysed in RIPA buffer^42^ and protein concentration was determined by BCA (ThermoFisher). Western blot was performed as previously described.^43^ Antibodies for Star-PAP (Abcam, Cambridge, UK), BIK (CST, Danvers, MA, USA), cytochrome *c* (CST), TOM20 (CST), Caspase 9 (CST) and *β*-tubulin (Sigma-Aldrich, St. Louis, MO, USA) were used according to the manufacturer's instructions.

### qPCR and RT-PCR

Total RNA was prepared with TRIzol (ThermoFisher) according to the manufacturer's protocol. Reverse transcription was performed using RevertAid H Minus First-Strand cDNA Synthesis Kit (ThermoFisher). For qPCR, SYBR Select Master Mix (ThermoFisher) was used with ABI 7500 Real-Time PCR System (ThermoFisher, Waltham, MA, USA). For RT-PCR, PrimeSTAR HS DNA Polymerase (Takara, Kusatsu, Japan) was used, and the products were analyzed in agarose gel. All primers were listed in Supplementary Materials.

### Clinical data set analysis

To investigate mRNA levels of Star-PAP, the public data sets E-TABM-157, GSE14548, GSE3744 and GSE9014 were analyzed in Oncomine (www.oncomine.org) according to the instructions. The online survival analysis software KM-plotter was employed for cancer survival analysis as previously described.^17^

### Apoptosis assay and *ΔΨ*m assay

Cell apoptosis was analyzed using Annexin V Apoptosis Detection Kit (BD, Franklin Lakes, NJ, USA) as previously described.^44^ JC-1 Mitochondrial Membrane Potential Assay Kit (Cayman, Ann Arbor, MI, USA) was used for *ΔΨ*m assay according to the manufacturer's manual and the method has been previously reported.^45^ FACS data were collected using BD FACSCalibur and analyzed using FlowJo (FlowJo LLC, Ashland, OR, USA).

### RNA immunoprecipitation

RIP was performed as reported with antibody against Star-PAP.^9^ A total of 10^7^ cells were used for preparation of mRNP lysate. Finally, RNA was purified with TRIzol (ThermoFisher) and reverse transcribed by RevertAid H Minus First Strand cDNA Synthesis Kit (ThermoFisher). Gene-specific primers were used for RT-PCR assay and products were analyzed by agarose gel. All primers are listed in Supplementary Materials.

### Soft agar assay

In six-well plate, 0.6% agar in growth medium was plated as the bottom layer, and then the top layer containing 2 000 cells and 0.3% agar in growth medium was plated. Fresh medium was added twice a week for 3 weeks. The colonies were visualized by Staining with Thiazolyl Blue Tetrazolium Bromide (Sigma-Aldrich) for 1 h at 37 °C.

### Xenografts in NOD/SCID mice

All animal procedures were conducted under the guidelines approved by the institutional animal care and use committee. Female NOD/SCID mice (Vital River, Beijing, China) were used as host for breast cancer xenografts. For tumor growth assay, the same amount (2 × 10^6^ in 20% matrigel) of control and Tet-On Star-PAP cells were incubated into the contralateral fourth inguinal mammary fat pad of NOD/SCID mice. The diet containing 0.02% doxycycline was supplied immediately, and tumor volume was measured weekly. For drug treatment experiment, NOD/SCID mice were incubated with 3 × 10^6^ cells as above. When tumor volume reached ∼50 mm^3^, doxorubicin (5 mg/kg) was administered i.v. twice a week, and the diet containing doxycycline was supplied in the course of treatment. Tumor was measured weekly and the volume was calculated as 1/2 × length × (width)^2^.

## Abbreviations

*ΔΨ*m: mitochondrial transmembrane potential
BIK: BCL2-interacting killer
RIP: RNA immunoprecipitation
EMT: epithelial–mesenchymal transition
