# Supplementary material for: Star-PAP, a poly(A) polymerase, functions as a tumor suppressor in an orthotopic human breast cancer model
Source: Cell Death Dis. 2017 Feb 2;8(2):e2582–. doi: 10.1038/cddis.2016.199 (PMC5386448; doi:10.1038/cddis.2016.199)
Supplement: Supplementary Figures [file cddis2016199x1.pdf]

**Supplementary Figure Legends**

**Figure S1.** Ectopic expression of Star-PAP inhibits growth of SUM-159PT cells. **(a)** Ectopic expression of Star-PAP was determined by western blot. β-Tubulin was used as the loading control. **(b)** SUM-159PT cells transfected with pCDNA3.1-Star-PAP were plated 5 000 cells/well, and colonies were stained with crystal violet. Representative images were shown (upper panel), and data from two replicate experiments were normalized and presented as mean ± SD (lower panel). $^{**}p < 0.01$. **(c)** Star-PAP inhibited proliferation of SUM-159PT cells. Cells were plated after transfection. Proliferation of cells was analyzed by MTS assay. Data were normalized to day 0. n=6, $^{**}p < 0.01$.

**Figure S2.** Star-PAP correlates with prognosis of breast cancer patients. KM-plotter was exploited to investigate the correlation between Star-PAP levels and clinical prognosis. Kaplan-Meier analysis of relapse-free survival of breast cancer patients stratified by the expression levels of Star-PAP was shown. Four major breast cancer subtypes were investigated. **(a)** Basal-like. **(b)** Her2-enriched. **(c)** Luminal A. **(d)** Luminal B. Number of patients, log-rank *p* value and hazard ratio (HR) were shown.

**Figure S3.** Doxycycline-induced overexpression of Star-PAP inhibits proliferation and induces apoptosis of MDA-MB-468 cells. **(a)** MDA-MB-468 Tet-On control and Star-PAP cells were treated with 200 ng/ml doxycycline. BIK mRNA level was detected by qPCR. Data from triplicate experiments were presented as mean ± SD. **(b)** Star-PAP inhibited proliferation of MDA-MB-468 cells. Tet-On control and Star-PAP cells were plated and induced with doxycycline immediately. Proliferation of cells was analyzed by MTS assay. Data were normalized to day 0. n=6, $^{**}p < 0.01$. **(c-d)** Doxycycline-induced overexpression of Star-PAP induced apoptosis of MDA-MB-468 cells. Tet-On control and Star-PAP cells were induced with doxycycline and analyzed by Annexin V/propidium Iodide Apoptosis Assay. Data from two replicate experiments were presented as mean ± SD. Dox, doxycycline.

**Figure S4.** Star-PAP sensitizes SUM-159PT cells to chemotherapy drugs. **(a-b)** SUM-159PT cells transfected with pCDNA3.1-Star-PAP or vector were treated with cisplatin and doxorubicin separately. Cell viability was measured by MTS assay. Dose-response curves were plotted. n = 6, error bar denotes SD. **(c-d)** SUM-159PT cells transfected with pCDNA3.1-Star-PAP or vector were treated with doxorubicin and DMSO, respectively. Apoptosis was analyzed by flow cytometry. Data from two replicate experiments were presented as mean ± SD. DXR, doxorubicin.

**Figure S5.** Star-PAP knockdown promotes proliferation of mammary epithelial cells MCF10A. **(a)** Knockdown of Star-PAP in MCF10A cells was examined by western blot. Two independent siRNAs were used for transfection. β-Tubulin was used as the loading control. **(b)** Knockdown of Star-PAP in MCF10A cells was examined by qPCR. Two independent siRNAs were used for transfection. mRNA levels were normalized to

control and presented as mean ± SD (n = 3). **(c)** MCF10A cells were plated in 96-well plates after transfected with two independent siRNAs. Proliferation of cells was analyzed by MTS assay, and data were normalized to day 0. n=5, $^{**}p < 0.01$, error bar denotes SD. **(d)** BIK mRNA was examined by qPCR after Star-PAP knockdown. Two independent siRNAs were used for transfection. mRNA levels were normalized to control and presented as mean ± SD (n = 3).
