# Supplementary material for: Star-PAP, a poly(A) polymerase, functions as a tumor suppressor in an orthotopic human breast cancer model
Source: Cell Death Dis. 2017 Feb 2;8(2):e2582–. doi: 10.1038/cddis.2016.199 (PMC5386448; doi:10.1038/cddis.2016.199)
Supplement: Supplementary Table 1 [file cddis2016199x2.pdf]

Supplementary Table S1. Sequences of siRNA

siRNA	sequence
siStar-PAP#1	AACUACGAGCTGCGAGAAA
siStar-PAP#2	GUGUGUUUGUCAGUGGCUU
shRNA	target sequence
shBIK#1	CCACACTTAAGGAGAACATAA
shBIK#2	TGGACGGTTTCACCACACTTA
shStar-PAP	AACUACGAGCTGCGAGAAA
