# Supplementary material for: Star-PAP, a poly(A) polymerase, functions as a tumor suppressor in an orthotopic human breast cancer model
Source: Cell Death Dis. 2017 Feb 2;8(2):e2582–. doi: 10.1038/cddis.2016.199 (PMC5386448; doi:10.1038/cddis.2016.199)
Supplement: Supplementary Table 2 [file cddis2016199x3.pdf]

**Table S2. Sequences of qPCR and RT-PCR primers**

| qPCR primer | sequence |
|---|---|
| Star-PAP forward | GAGTTCTTCCCTGGCTGTGT |
| Star-PAP reverse | AGCGATGGAGATTCTGGAGC |
| GAPDH forward | ACGACCACTTTGTCAAGCTCA |
| GAPDH reverse | TCTCTCTTCCTCTTGTGCTCT |
| BIK forward | CCCGACCATGGAGGTTCTT |
| BIK reverse | TGAGGCTCACGTCCATCTC |
| CDH1 forward | CGAGAGCTACACGTTCACGG |
| CDH1 reverse | GGGTGTCGAGGGAAAAATAGG |
| CDH2 forward | CAACGACGGGTTAGTCACCG |
| CDH2 reverse | CGGGTGCTGAATTCCCTTGG |
| FN1 forward | GAGGAAACCTGCTCCAGTGC |
| FN1 reverse | CACGAACATCGGTGAAGGGG |
| SNAI1 forward | ATGCACATCCGAAGCCACAC |
| SNAI1 reverse | TGCAGTGGGGACAGGAGAAG |
| SNAI2 forward | CGGACCCACACATTACCTTGT |
| SNAI2 reverse | AAAAAGGCTTCTCCCCCGTG |
| TWIST1 forward | GACCTAGATGTCATTGTTTCCAGA |
| TWIST1 reverse | CCCACGCCCTGTTTCTTTGA |
| VIM forward | CAACCTGGCCGAGGACATCA |
| VIM reverse | CGTGCCAGAGACGCATTGTC |
| | |
| RT-PCR primer | sequence |
| Star-PAP forward | GAGTTCTTCCCTGGCTGTGT |
| Star-PAP reverse | AGCGATGGAGATTCTGGAGC |
| GAPDH forward | ACGACCACTTTGTCAAGCTCA |
| GAPDH reverse | TCTCTCTTCCTCTTGTGCTCT |
| BIK forward | CCCGACCATGGAGGTTCTT |
| BIK reverse | TGAGGCTCACGTCCATCTC |
| HMOX-1 forward | ATGCCCCAGGATTTGTCAGA |
| HMOX-1 reverse | GAAGACTGGGCTCTCCTTGT |
