# Supplementary figures and images for: Star-PAP, a poly(A) polymerase, functions as a tumor suppressor in an orthotopic human breast cancer model
Source: Cell Death Dis. 2017 Feb 2;8(2):e2582–. doi: 10.1038/cddis.2016.199 (PMC5386448; doi:10.1038/cddis.2016.199)

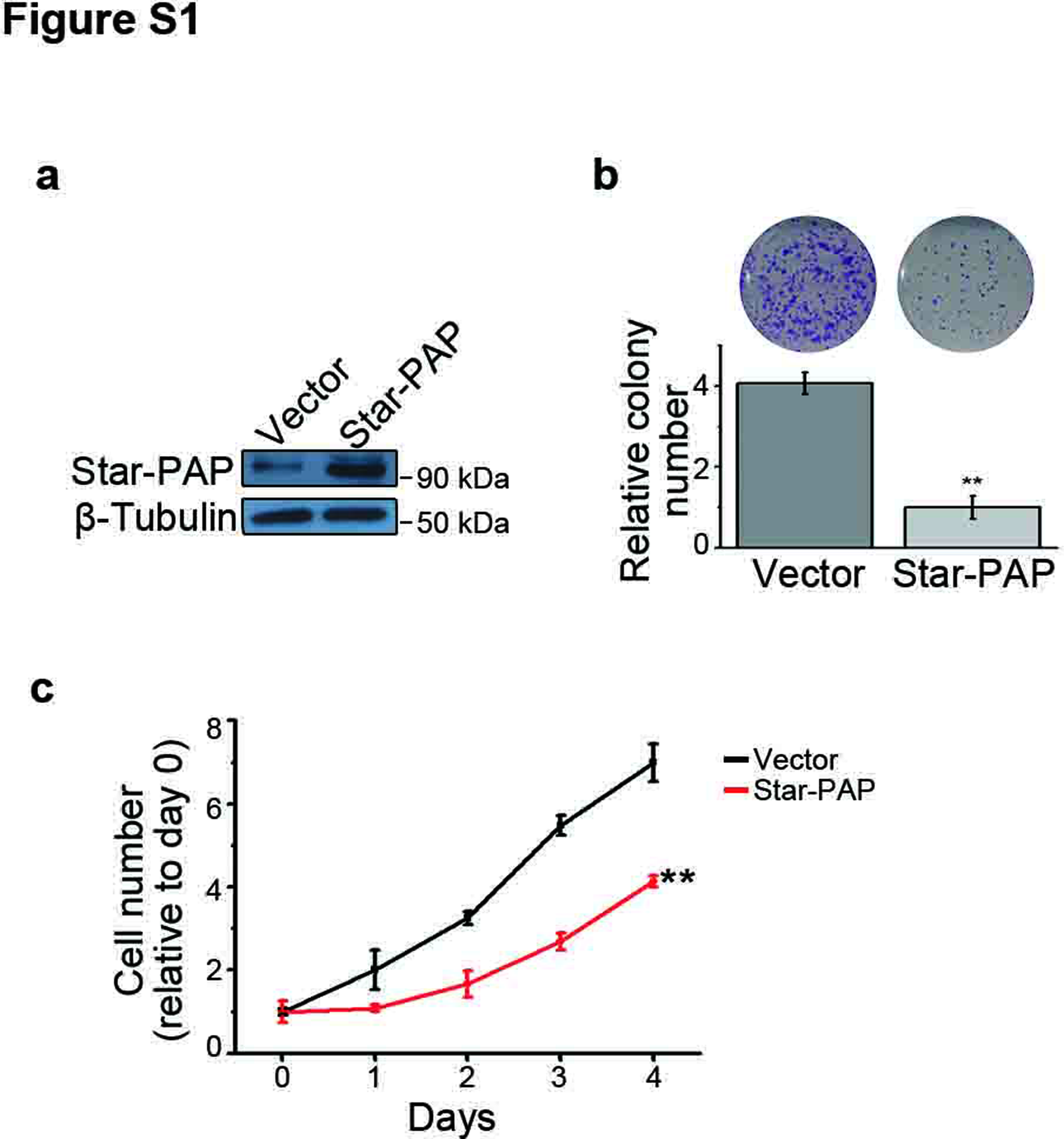

Supplement: Supplementary Figure S1 [file cddis2016199x4.tif]

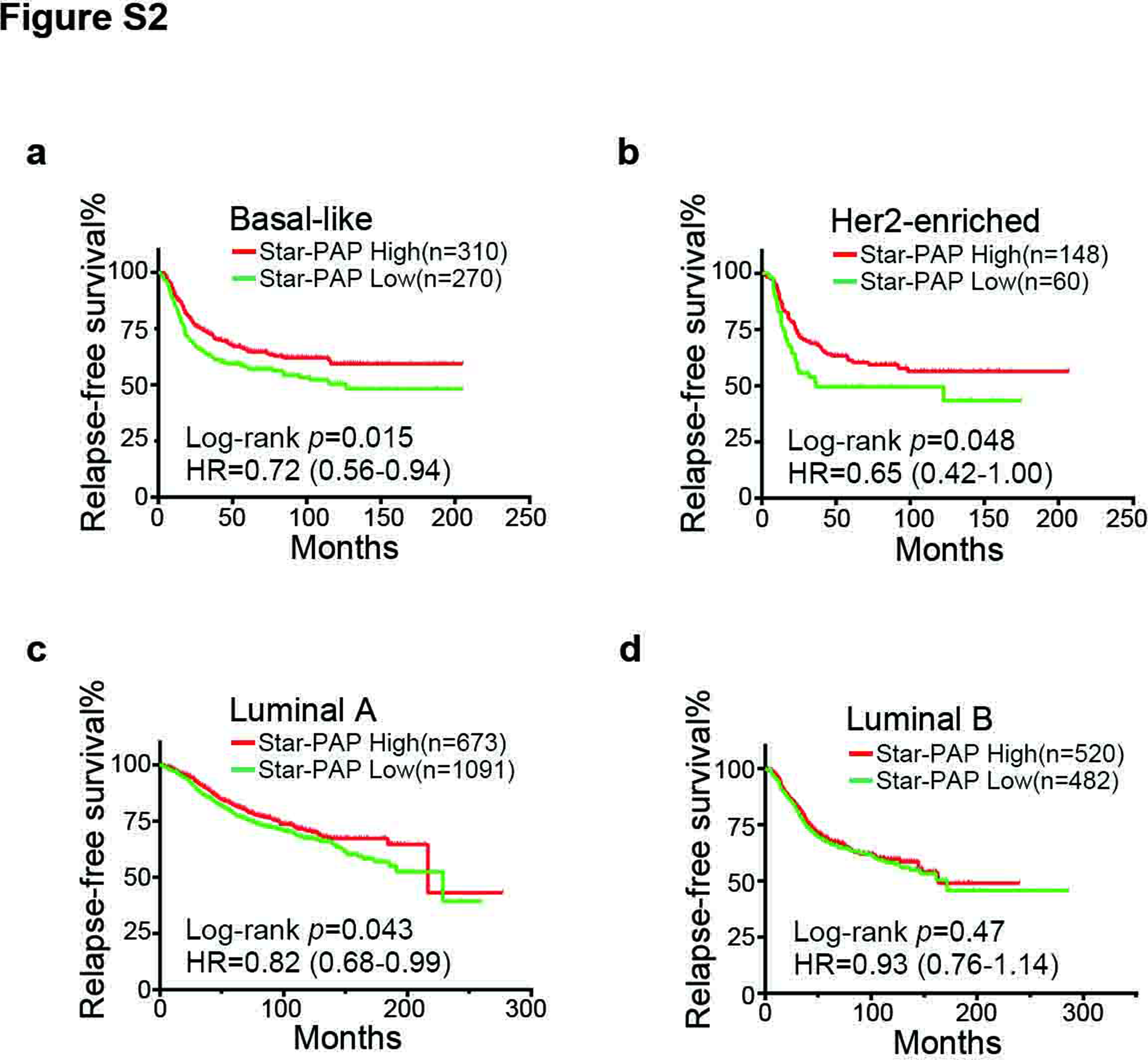

Supplement: Supplementary Figure S2 [file cddis2016199x5.tif]

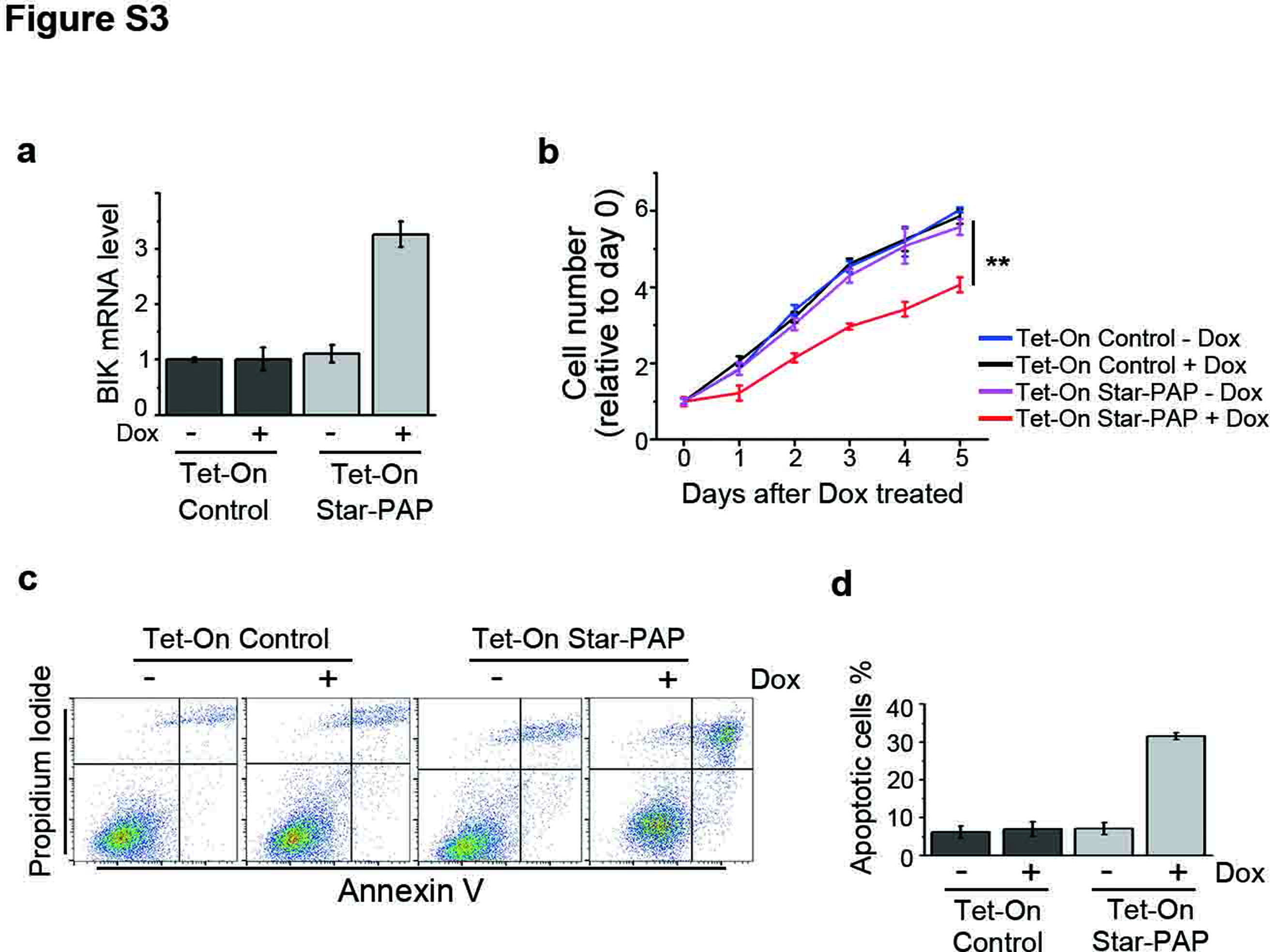

Supplement: Supplementary Figure S3 [file cddis2016199x6.tif]

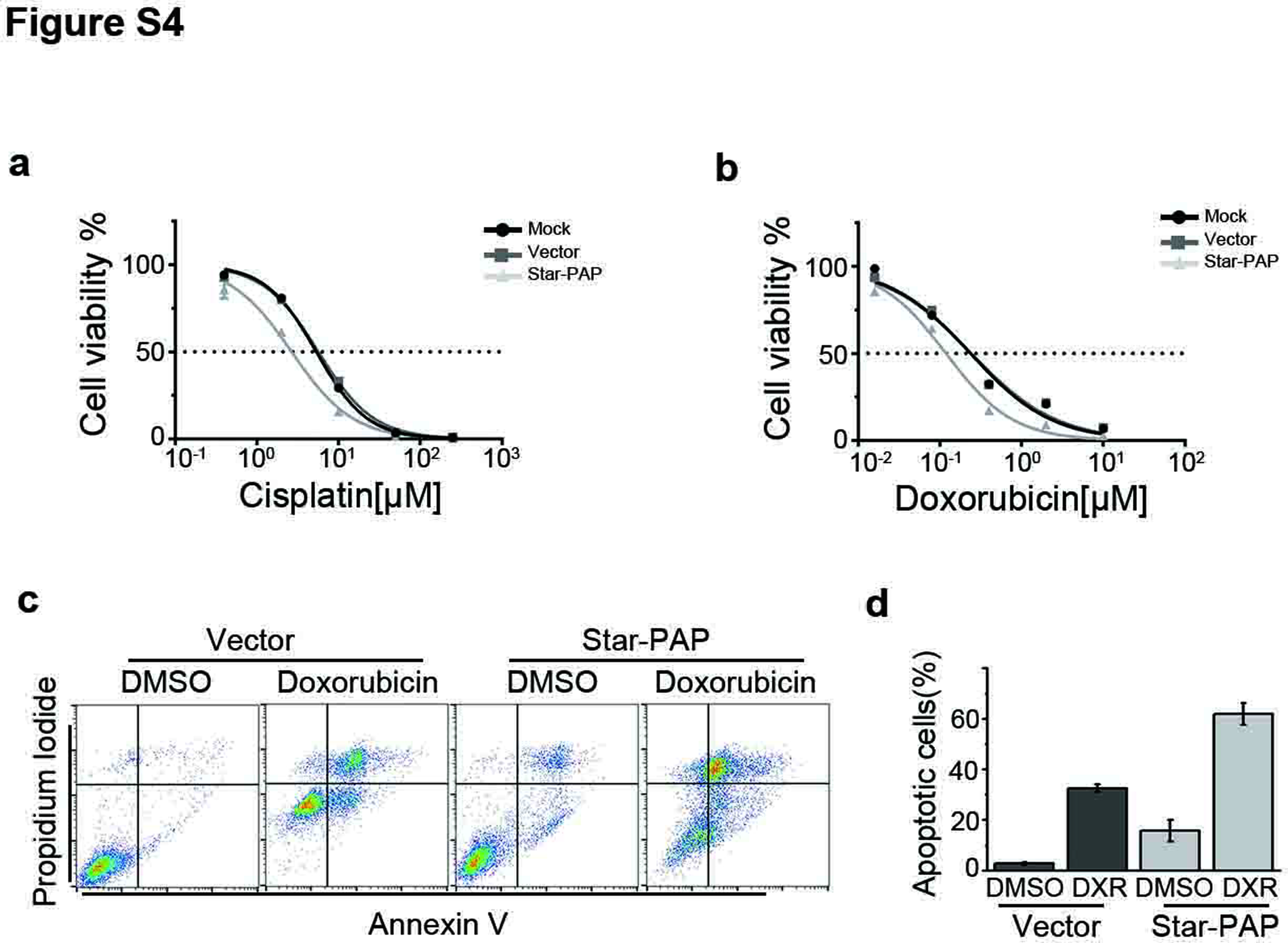

Supplement: Supplementary Figure S4 [file cddis2016199x7.tif]

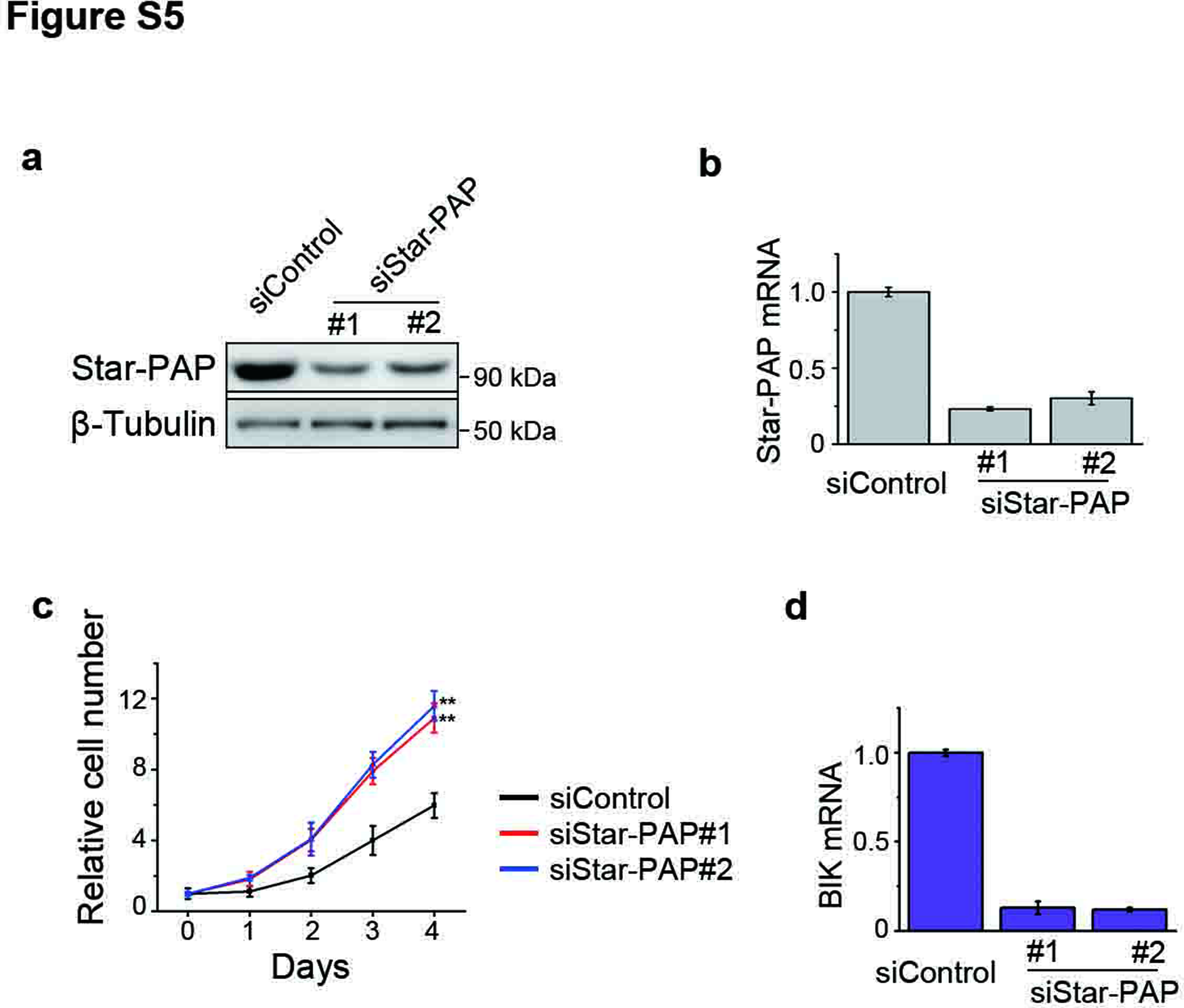

Supplement: Supplementary Figure S5 [file cddis2016199x8.tif]
